# Maintenance of Sugar-Sweetened Beverage Behaviors Among Adolescents and Their Caregivers: A Cluster Randomized Controlled Trial

**DOI:** 10.5888/pcd22.240520

**Published:** 2025-06-12

**Authors:** Annie Reid, Wen You, Theresa Markwalter, Kathleen Porter, Donna-Jean Brock, Philip Chow, Lee Ritterband, Jamie Zoellner

**Affiliations:** 1 UVA Cancer Center Research and Outreach Office, Department of Public Health Sciences, University of Virginia, Christiansburg; 2 Department of Public Health Sciences, University of Virginia, Charlottesville; 3 Department of Psychiatry and Neurobehavioral Sciences, University of Virginia, Charlottesville

## Abstract

The intake of sugar-sweetened beverages (SSBs) is a public health concern. Evidence-based behavior-change interventions can facilitate reductions in intake. Understanding maintenance of reductions after an initial intervention period is essential. In a cluster randomized controlled trial of 12 middle schools in Appalachia, we examined the 19-month maintenance effects of a 6-month school-based SSB reduction intervention tailored for middle school students and caregivers and demonstrated to be effective. Relative to their control counterparts, intervention students maintained significant reductions in SSB intake, while intervention caregivers did not show sustained effects. This study offers valuable insights into the long-term effect of school-based behavioral interventions designed to reduce intake of SSBs.

**Summary**

**What is already known on this topic?**

Behavior-change interventions to reduce intake of sugar-sweetened beverages (SSBs) have shown promising short-term outcomes, but little is known about the long-term maintenance effects of such interventions.

**What is added by this report?**

We report the 19-month maintenance effects of Kids SIP*smart*ER, a 6-month, multilevel, behavioral intervention that included classroom-based lessons for middle school students and a short-message-service (SMS) strategy for caregivers. Kids SIP*smart*ER led to significant reductions in SSB intake in students but not caregivers.

**What are the implications for public health practice?**

Study findings highlight the effect of Kids SIP*smart*ER in achieving sustained SSB reductions among rural students in Central Appalachia. More research is needed to explore how SMS interventions can sustain SSB reductions among caregivers.

## Objective

Sugar-sweetened beverages (SSBs) are a public health concern in the US; they have well-documented associations with many chronic health conditions (1). SSB intake is disproportionately high in Central Appalachia, where average intake is double the national average for adolescents and adults (2). Evidence-based SSB interventions are essential for addressing disparities in medically underserved rural areas, including interventions that can facilitate sustained behavior change.

The maintenance period of behavior-change interventions refers to outcomes observed 6 months or more after the last intervention contact (3). The literature examining the maintenance effects of behavior-change interventions (4), including the SSB-intake behaviors of adolescents, is limited. In 4 systematic reviews examining SSB intake, only 4 randomized controlled trials focused on middle school students and also included a maintenance period. Although these studies showed short-term reductions in SSB intake, none found improvements at long-term follow-up (5–8). Similarly, short-message-service (SMS) interventions also demonstrated positive outcomes, but none described long-term maintenance effects (9).

The Kids SIP*smart*ER intervention, the focus of this research, effectively decreased student and caregiver SSB intake at 7-month follow-up (2). The objective of this study was to test the hypothesis that improvements in SSB intake at 7 months among intervention participants would be sustained at 19 months.

## Methods

The Kids SIP*smart*ER cluster randomized controlled trial occurred from the 2018–2019 through the 2021–2022 academic school years in 12 middle schools in southwestern Virginia and West Virginia. Schools were randomized to the intervention or a delayed control. Protocol details are described elsewhere (10). All 7th grade students and 1 caregiver per student were eligible to participate, regardless of SSB intake. Students who assented to participate and whose parents consented for their student to participate were enrolled in the intervention and control; students completed a baseline assessment. All caregivers who enrolled in the intervention and control consented to participate and completed a baseline assessment. Enrolled students received a nominal prize, and enrolled caregivers received up to $30 in gift cards for survey completion. The study was approved by the University of Virginia Institutional Review Board.

Kids SIP*smart*ER was a multilevel, 6-month behavioral intervention targeting SSB intake among 7th-grade students and their caregivers. Students received 12 classroom-based lessons focused on skill-based health literacy concepts. A 2-way SMS strategy engaged caregivers in role modeling SSB behaviors, improving the home environment, and supporting rules and practices. Content was guided by the Theory of Planned Behavior and health literacy concepts. Maintenance data were collected at 19 months using the same survey used at baseline and 7-month follow-up. Participants self-reported demographic characteristics. SSB intake during the past month was assessed by using the validated Beverage Intake Questionnaire (11). Due to COVID-19 disruptions, student and caregiver paper-and-pencil surveys transitioned to an online format. Students completed surveys during a regular classroom period in 11 of 12 schools; 1 school conducted online surveys only due to closure.

We calculated changes in SSB intake in ounces per day from baseline to 19 months. Participants with change scores equal to or greater than twice the IQR were considered outliers and excluded from analysis (2). The IQR is good for identifying outliers, especially in skewed and asymmetric distributions (12). Our analyses of 19-month maintenance were identical to analyses at 7-month follow-up, whereby we used modified 2-part models with fixed effects to estimate between-group treatment effects over time for SSB outcomes (2). For the 19-month analyses, because of COVID-19 disruptions and concerns about nonrandom missing data, we used data only from participants who were present at follow-up, rather than an intention-to-treat approach, which was used at 7-month follow-up.

## Results

**Figure 1:**
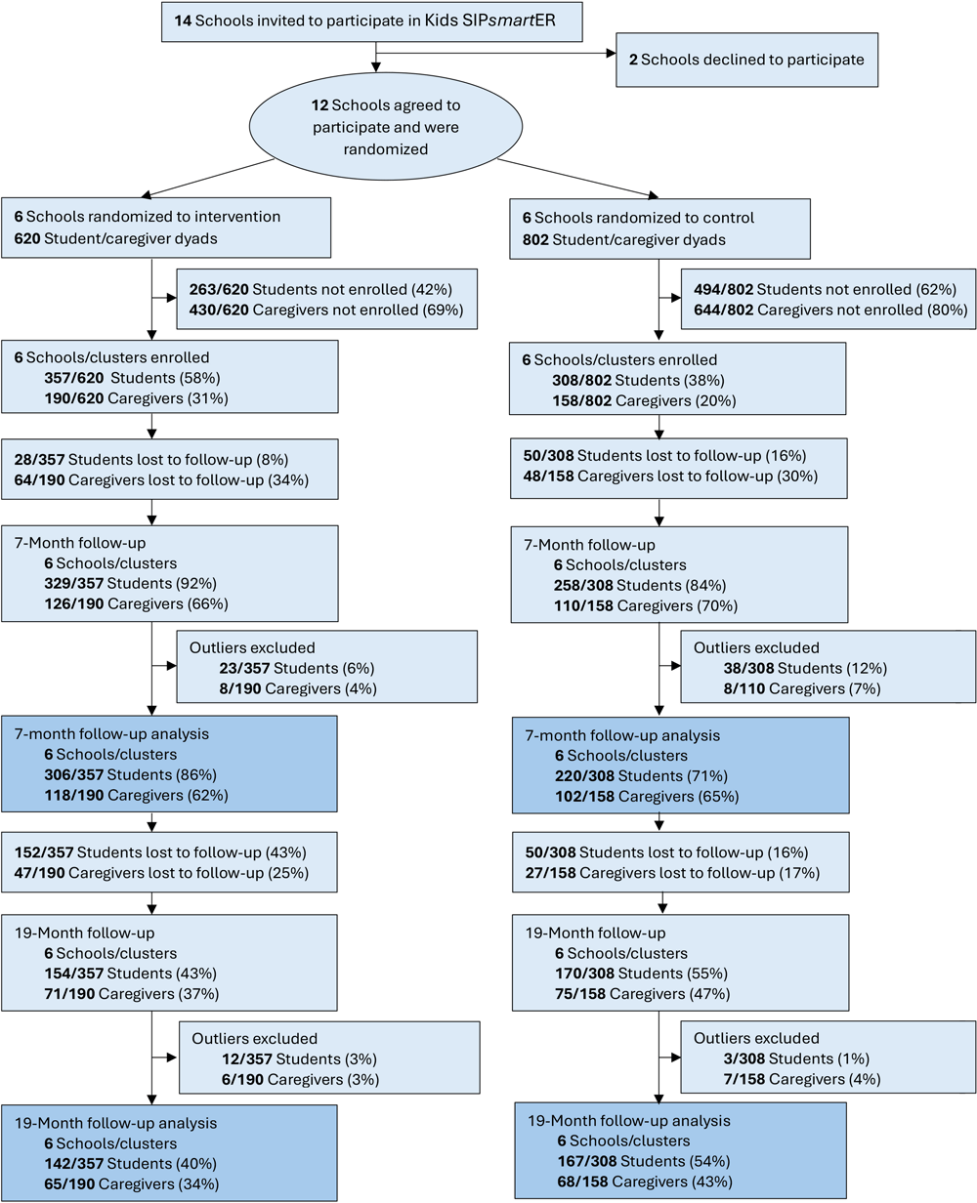
CONSORT (Consolidated Standards of Reporting Trials) flow diagram for Kids SIP*smart*ER, an intervention designed to decrease intake of sugar-sweetened beverages among middle school students and their caregivers in southwestern Virginia and West Virginia, 2018–2022.

Of the 526 students and 220 caregivers with complete data at 7-month follow-up, 324 (62%) students and 146 (66%) caregivers completed the maintenance assessment at 19 months (Figure 1 and Supplemental Table). The exclusion of outliers (15 [3%] students and 13 [6%] caregivers) resulted in 309 students and 133 caregivers for the analysis at 19 months. Of the 309 students who completed all 3 assessments, the mean age was 12.7 years, 54% were girls, 87% were White, and 48% had a healthy body mass index (Table).

**Table:** Baseline Demographic Characteristics of Enrolled Students and Caregivers Who Completed Baseline, 7-Month, and 19-Month Assessments, Overall and by Randomized Condition, Kids SIP*smart*ER

Characteristic	Overall	By randomized condition	
		Kids SIP*smart*ER^a^	Control
**Students**			
**Total no.**	309	142	167
**Age, mean (SD), y**	12.7 (0.5)	12.7 (0.6)	12.6 (0.4)
**Sex, no. (%)**			
Female	168 (54)	87 (61)	81 (49)
Male	135 (44)	53 (37)	82 (49)
Other or unknown	6 (2)	2 (1)	4 (2)
**Race, no. (%)**			
Black	16 (5)	6 (4)	10 (6)
White	268 (87)	124 (87)	144 (86)
Other or unknown	25 (8)	12 (8)	13 (8)
**Hispanic ethnicity, no. (%)**	10 (3)	4 (3)	6 (4)
**BMI^b^ *z*-score, mean (SD)**	0.9 (1.1)	0.9 (1.1)	0.9 (1.0)
**BMI^b^ percentile, mean (SD)**	72.9 (26.8)	72.4 (28.2)	73.3 (25.6)
**BMI^b^ categories, no. (%)**			
Underweight (BMI <5th percentile)	6 (2)	3 (2)	3 (2)
Healthy weight (BMI 5th to <85th percentile)	149 (48)	70 (49)	79 (47)
Overweight (BMI 85th to <95th percentile)	54 (17)	23 (16)	31 (19)
Obesity (BMI 95th to <99th percentile)	59 (19)	31 (22)	28 (17)
Severe obesity (BMI ≥99th percentile)	23 (7)	12 (9)	11 (7)
**Partial or complete parent participation, no. (%)**	169 (55)	80 (56)	89 (53)
**Caregivers**			
**Total no.**	133	65	68
**Age, mean (SD)**	41.7 (6.5)	42.4 (6.8)	41.1 (6.1)
**Sex, no. (%)**			
Female	127 (95)	63 (97)	4 (94)
Male	6 (5)	2 (3)	4 (6)
**Race, no. (%)**			
Black	3 (2)	1 (2)	2 (3)
White	126 (95)	62 (95)	64 (94)
Other or unknown	4 (3)	2 (3)	2 (3)
**Hispanic ethnicity, no. (%)**	0	0	0
**Education, no. (%)**			
High school diploma, GED, or less	28 (21)	13 (20)	15 (22)
Some college, associate degree	53 (40)	29 (45)	24 (35)
4-Year college degree or higher	48 (36)	22 (34)	26 (38)
Other or unknown	4 (3)	1 (1)	3 (4)
**Annual household income, no. (%)**			
<$25,000	17 (13)	11 (17)	6 (9)
$25,000-$49,999	26 (20)	8 (12)	18 (26)
$50,000-$74,999	24 (18)	15 (23)	9 (13)
≥$75,000	46 (35)	22 (34)	24 (35)
Other or unknown	20 (15)	9 (14)	11 (16)
**BMI, mean (SD)^c^**	30.8 (7.9)	31.2 (7.7)	30.4 (8.2)
**BMI categories, no. (%)**			
Underweight (BMI <18.5)	2 (1)	0	2 (3)
Healthy weight (BMI 18.5–24.9)	26 (20)	12 (18)	14 (21)
Overweight (BMI 25.0–29.9)	41 (31)	19 (29)	22 (32)
Obesity (BMI 30.0–34.9)	24 (18)	14 (22)	10 (15)
Severe obesity (BMI ≥35.0)	31 (23)	17 (26)	14 (21)
Other or unknown	9 (7)	3 (5)	6 (9)
**Weight, mean (SD)**			
In kilograms	85.1 (25.1)	85.5 (24.6)	84.7 (25.8)
In pounds	187.6 (55.4)	188.5 (54.2)	186.7 (56.9)

Abbreviations: BMI, body mass index, GED, General Educational Development.

a The Kids SIP*smart*ER cluster randomized controlled trial occurred from the 2018–2019 through the 2021–2022 academic school years in 12 middle schools in southwestern Virginia and West Virginia (10).

b Data available for 291 students.

c Calculated as weight in kilograms divided by height in meters squared.

**Figure 2:**
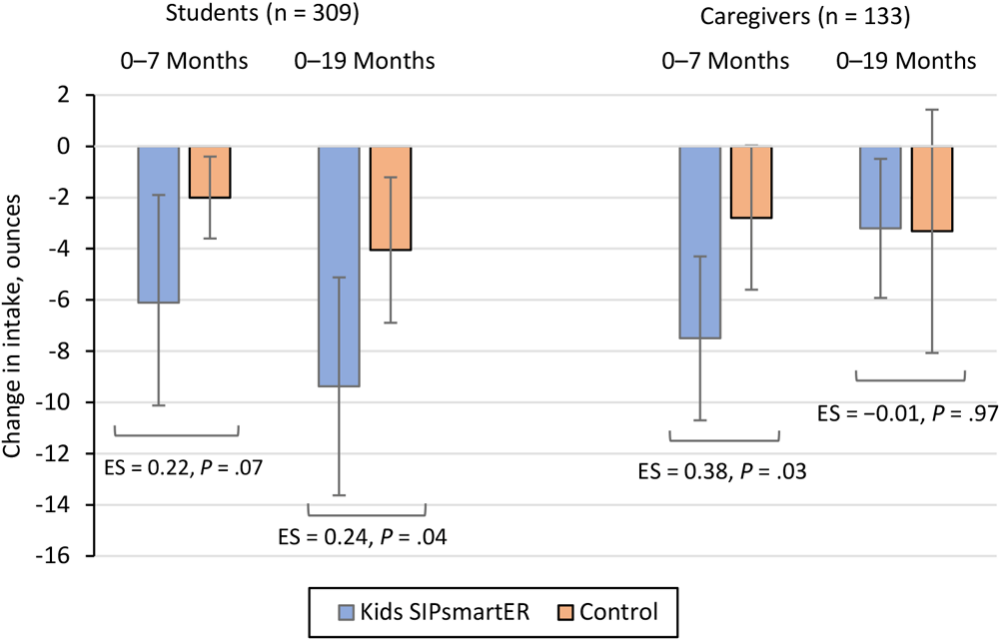
Changes in intake of sugar-sweetened beverages, in ounces, by randomized treatment condition, among students and caregivers in Kids SIP*smart*ER, an intervention designed to decrease intake among middle school students and their caregivers in southwestern Virginia and West Virginia, 2018–2022. Abbreviation: ES, effect size.

During the 6-month intervention, students who received the Kids SIP*smart*ER intervention significantly reduced their SSB intake by −6.1 ounces per day (95% CI, −10.1 to −1.9; *P* = .004), while control-group students reduced intake by −2.0 ounces per day (95% CI, −3.6 to −0.4; *P* = .01) (Figure 2). A relative difference of −4.0 ounces per day (95% CI, −8.4 to 0.3; *P* = .07; effect size [ES] = 0.22) favored the intervention group. In the 19-month period, intervention students decreased SSB intake by −9.4 ounces per day (95% CI, −13.6 to −5.1; *P* < .001), compared with a −4.0 ounce-per-day reduction in the control group (95% CI, −6.9 to −1.2; *P* = .005). Overall, between baseline and 19 months, we found a significant relative difference of −5.3 ounces per day (95% CI, −10.4 to −0.2; *P* = .04; ES = 0.24), indicating that intervention students maintained their SSB improvements at 19 months.

**Table:** 

Condition	Students, no. of ounces		Caregivers, no. of ounces	
	0–7 Months	0–19 Months	0–7 Months	0–19 Months
Kids SIP*smart*ER	−6.1 (−10.1 to −1.9)	−9.4 (−13.6 to −5.1)	−7.5 (−10.7 to −4.3)	−3.2 (−5.9 to −0.5)
Control	−2.0 (−3.6 to −0.4)	−4.0 (−6.9 to −1.2)	−2.8 (−5.6 to 0.03)	−3.3 (−8.1 to 1.4)

During the 6-month intervention period, caregivers in the intervention group significantly reduced their SSB intake by −7.5 ounces per day (95% CI, −10.7 to −4.3; *P* < .001), while caregivers in the control group reduced intake by −2.8 ounces per day (95% CI, −5.6 to 0.03; *P* = .05) (Figure 2). A relative difference of −4.7 ounces per day (95% CI, −8.9 to −0.3; *P* = .03; ES = 0.38) favored intervention caregivers over those in the control group. In the 19-month period, intervention caregivers reduced SSB intake by −3.2 ounces per day (95% CI, −5.9 to −0.5; *P* = .02), while caregivers in the control group decreased intake by −3.3 ounces per day (95% CI, −8.1 to 1.4; *P* = .17). The nonsignificant between-group effect (*P* = .97; ES = −0.01) indicates that intervention caregivers did not maintain their SSB improvements at 19 months.

## Discussion

Relative to students in the control group, students in the Kids SIP*smart*ER intervention maintained improved SSB-intake behaviors at 19 months. To our knowledge, this is the first behavioral intervention to show maintenance of SSB-intake behaviors among adolescents beyond the initial behavioral intervention period (5–8). While the overall between-group ES was relatively small (ie, 0.24), our intervention was intended for all adolescents regardless of baseline SSB intake. From this perspective, primary prevention interventions with small effects can have important public health implications. The sustained 9-ounce SSB reduction among adolescents receiving the Kids SIP*smart*ER intervention has clinical relevance, as epidemiologic studies consistently link each 8-to-12–ounce decrease in SSB intake to health benefits (1).

Contrary to our hypothesis, the significant effects among intervention caregivers enrolled in the SMS intervention at 7-month follow-up were not sustained at 19 months. This is a key contribution to the field, because little is known about the maintenance effects of SMS interventions. In a meta-analysis of 35 SMS studies, only 7 collected data after a no-intervention maintenance period (9). The pooled maintenance effect from these studies was small but significant (Cohen *d* = 0.17; *P* = .02); however, none focused on nutrition. Our contrasting findings highlight the need to better understand strategies for sustaining behavior change, including changes in SSB intake, in “low-touch” interventions (ie, interventions, such as SMS interventions, that maximize the use of technology while minimizing direct human interaction to engage audiences).

Our study has 2 key limitations. First, the amount of missing data, largely due to COVID-19, should be considered when interpreting findings. Notably, relative to the 7-month postintervention period in this maintenance sample, the primary outcomes of the 7-month postintervention period, which included a larger sample and used an intention-to-treat analysis, revealed some differences in effect sizes for students (ES = 0.35 vs 0.22) and caregivers (ES = 0.33 vs 0.38) (10). Second, our study may lack generalizability beyond the unique rural Central Appalachian region. Despite these limitations, our study’s strengths include a rigorous cluster randomized controlled trial design, use of validated SSB measures, and a focus on the rural medically underserved Appalachian region.

Our findings demonstrate that Kids SIP*smart*ER — a school-based curriculum grounded in the Theory of Planned Behavior and health literacy — effectively maintains SSB reductions among adolescents in medically underserved rural areas. Given that even modest reductions in SSB intake are linked to health improvements, this study highlights the potential effect of disseminating Kids SIP*smart*ER to achieve sustained reductions in SSB intake among rural students. However, caregivers did not maintain reductions in SSB intake, underscoring the need for strategies to ensure lasting behavior change in low-touch SMS interventions or other scalable approaches.

## Appendix

**Supplemental Table:** Study Enrollment and Retention by Randomized Condition, Kids SIP*smart*ER^a^

Stage of intervention	Intervention	Control
**Determined to be eligible for enrollment**		
No. of dyads	620	802
No. of dyads per school/cluster, median (range)	97 (63–172)	137 (77–164)
**Reasons for not enrolling**		
Students		
No parental consent	234	462
Parental consent, but no student assent	19	20
Parental consent and student assent, but no baseline data	10	21
All reasons, no./total (%)	263/620 (42)	494/802 (62)
Caregivers		
No consent	391	573
Consent, but no baseline data	39	71
Both reasons, no./total (%)	430/620 (69)	644/802 (80)
**Enrollment**		
Students		
No. of students	357	308
Median (range) per school/cluster	57 (23–117)	51 (38–68)
Caregivers		
No. of caregivers	190	158
Median (range) per school/cluster	30 (10–71)	23 (15–46)
**7-Month follow-up (after excluding those with no 7-month data)**		
Students		
No. of students	329	258
Median (range) per school/cluster	50 (21–113)	39 (33–60)
Caregivers		
No. of caregivers	126	110
Median (range) per school/cluster	18 (5–54)	17 (11–30)
**Included in 7-month analysis (after excluding outliers)**		
Students		
No. of students	306	220
Median (range) per school/cluster	46 (21–108)	35 (29–46)
Caregivers		
No. of caregivers	118	102
Median (range) per school/cluster	17 (5–49)	17 (10–25)
**19-Month follow-up (after excluding those with no 19-month data)**		
Students		
No. of students	154	170
Median (range) per school/cluster	27 (10–39)	27 (15–42)
Caregivers		
No. of caregivers	71	75
Median (range) per school/cluster	11 (4–27)	13 (7–18)
**Included in 19-month analysis (after excluding outliers)**		
Students		
No. of students	142	167
Median (range) per school/cluster	26 (10–36)	26 (15–42)
Caregivers		
No. of caregivers	65	68
Median (range) per school/cluster	9 (4–26)	11 (6–17)

^a^ The Kids SIP*smart*ER cluster randomized controlled trial occurred from the 2018–2019 through the 2021–2022 academic school years in 12 middle schools in southwestern Virginia and West Virginia (10).
